# Hypermethylated Epidermal growth factor receptor (EGFR) promoter is associated with gastric cancer

**DOI:** 10.1038/srep10154

**Published:** 2015-05-11

**Authors:** Xiaoling Weng, Hong Zhang, Junyi Ye, Mengyuan Kan, Fatao Liu, Ting Wang, Jiaying Deng, Yanfang Tan, Lin He, Yun Liu

**Affiliations:** 1 Institutes of Biomedical Sciences, Fudan University, Shanghai 200032, PR China; 2 Institute for Nutritional Sciences, Shanghai Institutes for Biological Sciences, Chinese Academy of Sciences, Shanghai 200031, PR China; 3 Department of Radiation Oncology, Fudan University Shanghai Cancer Center, 270 Dongan Road, Shanghai 200032, PR China; 4 Bio-X Center, Key Laboratory for the Genetics of Developmental and Neuropsychiatric Disorders, Ministry of Education, Shanghai Jiao Tong University, Shanghai 200030, PR China; 5 Key Laboratory of Molecular Medicine, Ministry of Education, Department of Biochemistry and Molecular Biology, Fudan University Shanghai Medical College, Shanghai 200032, PR China

## Abstract

Epidermal growth factor receptor (EGFR) is a member of the receptor tyrosine kinases ErbB family and it is found to be overexpressed in gastric cancer. However, the mechanism of the regulation of the EGFR expression is still unknown. We used the Sequenom EpiTYPER assay to detect the methylation status of the EGFR promoter in normal and tumour tissues of 30 patients with gastric cancer. We also carried out quantitative real time PCR (qPCR) to detect the expression level of EGFR in our 30 patients. Notably, increased methylation level at EGFR promoter was found in tumour tissues than the corresponding adjacent noncancerous. In both Region I DMR and Region II DMR detected in our study, tumor tissues were significantly hypermethylated (P = 2.7743E−10 and 2.1703E−05, respectively). Region I_⊿CpG_2 was also found to be associated with the presence of distant metastasis (P = 0.0323). Furthermore, the results showed a strongly significant association between the relative EGFR expression and the EGFR methylation changes in both Region I and Region II (P = 0.0004 and 0.0001, respectively). Our findings help to indicate the hypermethylation at EGFR promoter in gastric cancer and it could be a potential epigenetic biomarker for gastric cancer status and progression.

Gastric cancer is the third most common malignant tumor and the second most frequent cause of cancer death worldwide^1^. Despite that tremendous efforts have been made in chemotherapy, radiotherapy, and surgical techniques, the survival rate of patients with advanced gastric cancer is still low^2^. Nowadays, in gastric cancer, the molecular mechanisms underlying tumorigenesis, proliferation, progression and drug resistance have been studied and it is necessary to find more diagnostic markers which contribute to gastric cancer.

EGFR belongs to the family of receptor tyrosine kinases (RTK) ErbB, which consisting of HER1/EGFR/ErbB1, HER2/Neu/ErbB2, HER3/ErbB3 and HER4/ErbB4^3^. EGFR is overexpressed in various cancers, including non-small cell lung cancer^4^, colorectal cancer^5^, pancreatic cancer^6^, esophagogastric cancer^7^ and gastric cancer^8^ as well. High expression level of EGFR is associated with an increased risk of invasion or metastasis; while the inhibition of EGFR leads to decreased cancer cell division, migration, angiogenesis and apoptosis in solid tumors^9^.

EGFR expression in cancer cells is tightly controlled, but the mechanism of the regulation of the EGFR expression is not fully studied. Epigenetic regulation is a biological mechanism by which gene expression is modulated through DNA methylation and histone modifications. DNA methylation is among the best studied epigenetic modifications and the methylation of cytosine at CpG dinucleotides is an important regulatory modification throughout the genome^10^. Understanding of the mechanisms underlying promoter methylation status and the regulation of EGFR expression might lead to the development of useful clinical biomarkers.

In our study, we used the Sequenom EpiTYPER assay to study the relationship between the methylation changes of the EGFR promoter and gastric cancer as well as its clinical characteristics such as histology differentiation, histologic grading, infiltration, TNM stage, and distant metastasis. We also carried out quantitative real time PCR (qPCR) to detect the expression level of EGFR to see the relationship between the EGFR methylation changes and the relative EGFR expression. We aimed to investigate whether methylation status of the EGFR promoter correlates with malignancy and patient outcome in gastric cancer.

## Methods

### Subjects

We analyzed 30 pairs of tissues (gastric cancer tissues and corresponding noncancerous tissues) from surgically removed primary gastric cancer in Ruijin Hospital of Shanghai Jiaotong University School of Medicine between July 2011 and May 2013. All participants were Han Chinese in origin and were examined histopathologically to confirm the diagnosis. All patients (23 males and 7 females, mean age 64.5 years, range 42–81 years) were at initial presentation and had no radiotherapy or chemotherapy history before surgery. Control tissues were the corresponding non-cancerous mucosa from the stomach of cancer patients, and excised beyond 5–7 cm from neoplastic lesions. The tissue samples were immediately frozen in liquid nitrogen and stored at −80 °C until analysis. A standard informed consent was established and all the participants signed the consent. The study protocol was approved by the ethics committee of the Shanghai Institute for Biological Sciences, and the methods were carried out in accordance with the approved guidelines.

### DNA methylation analysis

Genomic DNA was isolated from 25 ug tissue samples using the QIAamp DNA Mini Kit following the manufacturer’s protocol (QIAGEN, Hilden, Germany) and a Thermo NanoDrop2000 (Thermo, Wilmington, USA) was used to detect 260/280 nm UV absorbance ratio and concentration. Bisulfite conversion of DNA was carried out using the Epitect Bisulfite Kit (QIAGEN, Hilden, Germany).

**Figure 1:**
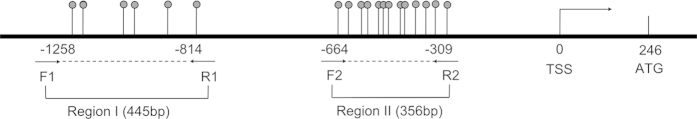
Schematic representation of the two regions of the EGFR promoter. The successful genotyping CpG sites are indicated with lollipop markers and some CpG sites were detected together. The forward and reverse primers are shown with arrows below the diagram. TSS: transcription initiation site.

Quantitative methylation analysis of DNA was performed using MassARRAY EpiTYPER assays (Sequenom, San Diego, CA). Two regions of the EGFR promoter were detected in our study (Fig. 1). Primers designed by Epidesigner (Sequenom, San Diego, CA; http://www.epidesigner.com) were as follows: Region I-F: 5′- aggaagagagGGGTAGTGAGTAGATTTGTGTTTGTT-3′, Region I-R:5′- cagtaatacgactcactatagggagaaggctATATCCCACTACCCCTATAACTCCC-3′; Region II-F:5′- aggaagagagGGAGTTGGGTGTTTTTATTTTAGATG-3′, Region II-R:5′- cagtaatacgactcactatagggagaaggctTACAAACCCAACCTATATCCAAATC-3′. Polymerase chain reaction (PCR) amplification was performed using a 5 ul reaction mixture and followed by SAP cleanup and T Cleavage. 20 ul H2O and 6 mg of Clean Resin (Sequenom, San Diego, CA) were added to the T Cleavage transcription products to remove bilvalent cation adducts. The samples were then transferred to a SpectroCHIP® array and sequenced on a MassARRAY analyzer (Sequenom, San Diego, CA). The amplicon comprised 26 CpG sites (8 of Region I and 18 of Region II ) were located in: Human Genome 19 assembly – chr7: 55,085,467-55,085,911 and chr7: 55,086,061-55,086,416 (GRCh37/hg19), Region I_CpG_1: 55,085,495; Region I_CpG_2: 55,085,520; Region I_CpG_3: 55,085,538; Region I_CpG_4: 55,085,661; Region I_CpG_5: 55,085,708; Region I_CpG_6: 55,085,860; Region I_CpG_7: 55,085,862; Region I_CpG_8: 55,085,886; Region II_CpG_1: 55,086,091; Region II_CpG_2: 55,086,122; Region II_CpG_3: 55,086,142; Region II_CpG_4: 55,086,146; Region II_CpG_5: 55,086,162; Region II_CpG_6: 55,086,164; Region II_CpG_7: 55,086,166; Region II_CpG_8: 55,086,172; Region II_CpG_9: 55,086,175; Region II_CpG_10: 55,086,210; Region II_CpG_11: 55,086,221; Region II_CpG_12: 55,086,248; Region II_CpG_13: 55,086,269; Region II_CpG_14: 55,086,272; Region II_CpG_15: 55,086,285; Region II_CpG_16: 55,086,288; Region II_CpG_17: 55,086,304; Region II_CpG_18: 55,086,391. The data for each CpG site or aggregates of multiple CpG sites were analysed using EpiTyper Software (Sequenom, San Diego, CA). And 10 DNA samples from 5 patients were randomly selected to be replicated in this study on the same bisulfite-converted sample, and yielded a highly consistent result (R^2^ = 0.95). Experiment on two independent bisulfate-converted samples of 5 patients was also yielded a highly consistent result (R^2^ = 0.92).

### EGFR expression analysis

RNA was extracted from fresh, frozen tissue using RNA isolation reagents following the manufacturer’s protocol (TRIzol, Invitrogen, Carlsbad, CA). cDNA synthesis was performed using SuperScript® III First-Strand Synthesis Kits according to the manufacturer’s protocol (Invitrogen, Carlsbad, CA).

Real-time Quantitative PCR was performed on an ABI VIIA^TM^ 7 Thermal Cycler (Life Technologies, Carlsbad, California, USA). The primers F: 5′-AGGCACGAGTAACAAGCTCAC-3′ and R: 5′-ATGAGGACATAACCAGCCACC-3′ were designed for the EGFR with 177 bp products. The primers F: 5′-GGAGCGAGATCCCTCCAAAAT-3′ and R: 5′-GGCTGTTGTCATACTTCTCATGG-3′ were designed for the GAPDH with 197 bp products. The PCR reactions included 2 × SYBR Green SuperReal PreMix Plus (TIANGEN, Shanghai, China), 10 nM forward and reverse primer and 5 ul 80-times diluted cDNA template. Cycling conditions for all primer pairs were 10 min at 95 °C, followed by 40 cycles of 15 s at 95 °C and 1 min at 60 °C. All samples were performed in duplicate to capture intra-assay variability, and multivariate samples were randomly chosen to test the reproducibility of the assay. All of the samples were successfully detected and showed a coefficient of variation (CV)<1%. The expression of GAPDH was used to normalize that of the EGFR gene, and the expression level of EGFR was expressed as 2^−⊿⊿Ct^, where ⊿Ct = Ct (EGFR)-Ct (GAPDH), ⊿⊿Ct =⊿Ct (Tumour)−⊿Ct (Normal). The relative expression level was 1 in control tissue, while it was the value of 2^−⊿⊿Ct^ in tumour tissue.

### Statistical analysis

The statistical analyses were performed using the R program ( http://www.r-project.org/). Student paired t-tests were used for the comparison of EGFR expression and methylation changes between primary tumors and adjacent noncancerous tissues. We also used the Wilcoxon signed-rank test to do the comparisons of paired tissues. The association of methylation changes as well as EGFR expression and clinical parameters were detected using linear regression analysis. Spearman correlation test was also used for the correlation analysis of participant characteristics and the methylation differences in DMR I and DMR II. A P value<0.05 was considered significant in these analyses.

## Results

### Population Characteristics

The clinical characteristics of the patients are summarized in Table 1. Of the 30 patients, 17 patients had well or moderate histology differentiation and 13 patients presented with low histology differentiation. Distant metastasis was detected in 3 patients and 26 patients were classified with grade III or IV TNM stage.

### Methylation status of EGFR promoter and its relationship with clinical pathological factors

**Figure 2:**
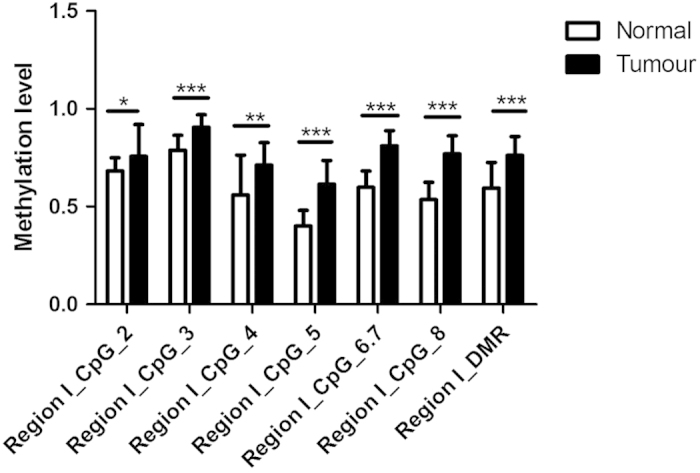
Average methylation levels of EGFR promoter in both normal and tumour tissues (Region I). Data are shown as mean ± SD (*P < 0.05, **P < 0.01, ***P < 0.001).

**Figure 3:**
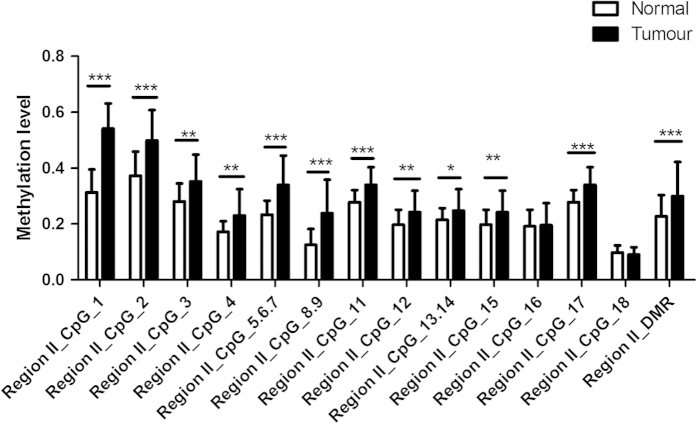
Average methylation levels of EGFR promoter in both normal and tumour tissues (Region II). Data are shown as mean ± SD (*P < 0.05, **P < 0.01, ***P < 0.001).

The EGFR promoter methylation levels were detected at 7 CpG sites of Region I and 17 CpG sites of Region II (Table 2). Tumour tissues were hypermethylated in more than 90% GC patients in both two regions. Methylation levels were analyzed in matched pairs, 7 out of 8 Region I CpG sites (CpG2, CpG3, CpG4, CpG5, CpG6.7 and CpG8) (Fig. 2) and 15 out of 18 Region II CpG sites (CpG1, CpG2, CpG3, CpG4, CpG5.6.7, CpG8.9, CpG11, CpG12, CpG13.14, CpG15 and CpG17) (Fig. 3) showed significant differences in methylation status between tumor and adjacent noncancerous tissues. Tumor tissues were significantly hypermethylated in both Region I DMR and Region II DMR (P = 2.7743E−10 and 2.1703E−05, respectively).

For the clinical characteristics, the evaluated categories were age, gender, histology differentiation, histology, infiltration, TNM stage and distant metastasis. The average methylation differences in DMR I and DMR II were not significantly correlated with the clinical factors (Table 3). As shown in Table 4, Region I_⊿CpG_2 was found to be associated with the presence of distant metastasis (P = 0.0323). However, other CpG mehylation changes were not significantly correlated with the clinical factors.

### EGFR expression and its relationship with clinical pathological factors

**Figure 4:**
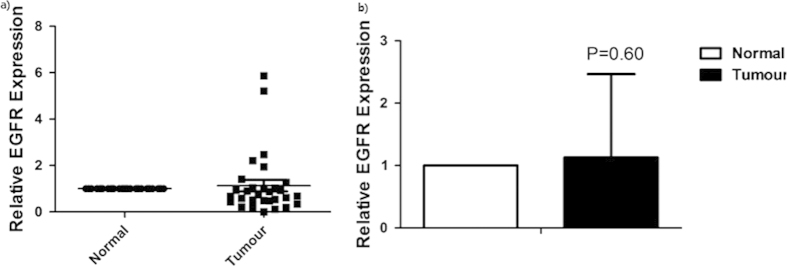
EGFR expression levels between tumor and normal tissues in 30 GC patients. **a**) Relative EGFR Expression data. **b**) Comparisons of Relative EGFR Expression data (P = 0.60). Data are shown as mean ± SD.

We used Quantitative Real-Time PCR to detect the expression changes in 30 patients. The EGFR was overexpressed in 33.3% (10 out of 30) patients, which is consistent with the established knowledge of EGFR overexpression in ~30% of GC patients^11^ (Fig. 4a). However, it was not highly differentially expressed among the 30 patients (normal and tumour, 1 and 1.13, respectively, P = 0.60) (Fig. 4b). Linear regression analysis showed that relative EGFR expression was not significantly correlated with the clinical factors (P > 0.05).

### Association of EGFR expression and EGFR promoter methylation changes

**Figure 5:**
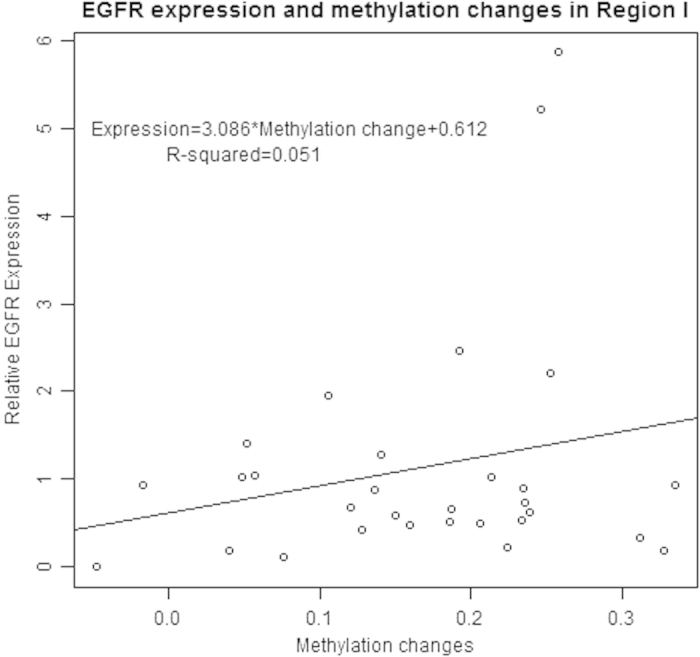
Association of EGFR expression and EGFR promoter methylation changes in Region I.

**Figure 6:**
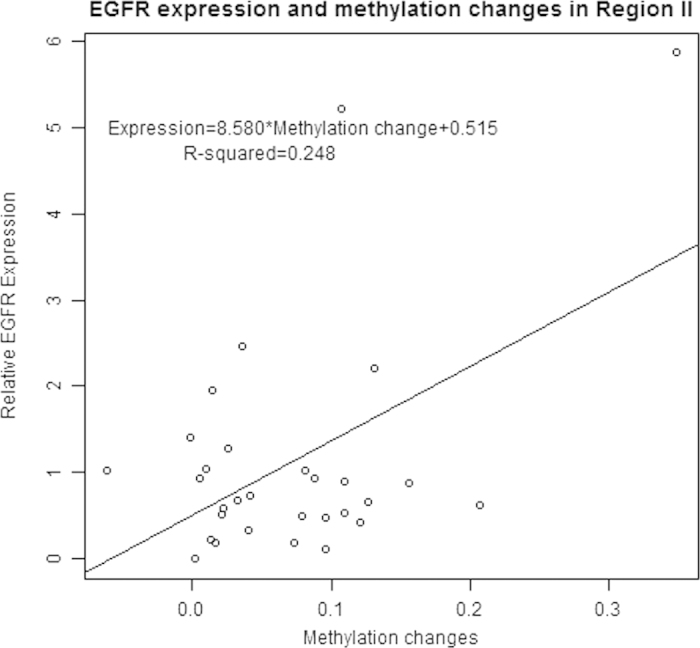
Association of EGFR expression and EGFR promoter methylation changes in Region II.

**Figure 7:**
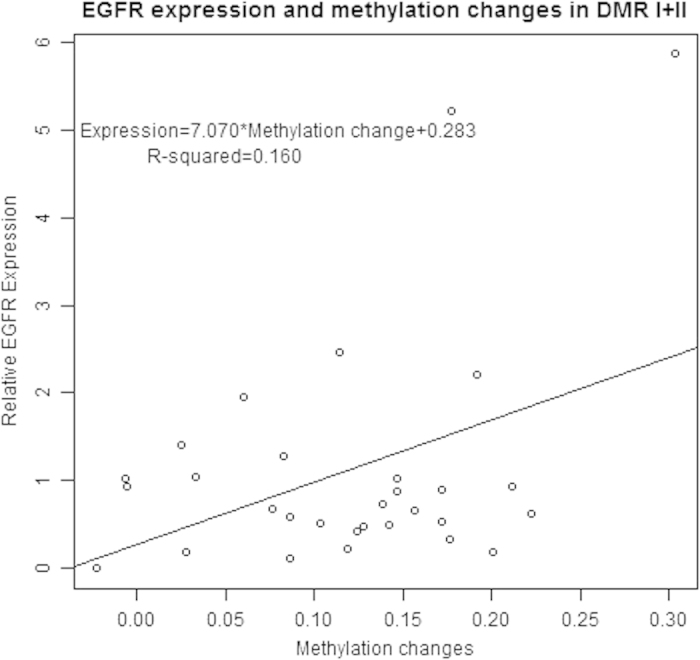
Association of EGFR expression and EGFR promoter methylation changes in DMR I + II.

Combined the data of EGFR methylation and expression, we found the EGFR expression was significantly associated with the EGFR methylation status in both Region I (P = 0.0004, Fig. 5) and Region II (P = 0.0001, Fig. 6) as well as DMR (I + II) (P = 0.0002, Fig. 7). The relative EGFR expression was significantly positively associated with the methylation changes.

## Discussion

Epidermal growth factor receptor regulates diverse functions in normal cells and plays a critical role in a wide range of human cancers^12^,^13^. EGFR is recognized as oncogenic driver in tumorigenesis and a target for cancer therapies^14^,^15^,^16^. Evidence suggests that mutations in EGFR^17^,^18^, and inframe deletions or gene amplifications^19^,^20^ are associated with aberrant expression of EGFR. To date, it is important to understand other key regulators leading to the overexpression of EGFR in various cancers and one promising mechanism is epigenetics^21^.

EGFR overexpression was detected in 27.4% of gastric cancer samples in a large case series^11^ and it is likely to be an independent predictor of poor prognosis^22^,^23^. Higher expression of EGFR in gastric cancer is also associated with increased risk of recurrence^24^, poor differentiation, higher stage disease, and large tumor size^25^. So it is of great value to determine EGFR status to interpret future clinical trials properly using EGFR targeted agents. And EGFR promoter methylation may be an important epigenetic regulation for EGFR expression and may be an epigenetic biomarker.

In colorectal cancer, EGFR promoter hypermethylation made worse median progression-free survival and worse median overall survival^26^. In gliomas, promoter hypermethylation of EGFR may play a role in progression of gliomas^27^. To the best of our knowledge, few researches are available yet to describe the alteration in methylation status at the promoter of EGFR in gastric cancer. The aim of this study is to investigate the methylation status at CpG sites of the promoter of EGFR in gastric cancer tissues and corresponding noncancerous tissues. We also aimed to investigate the epigenetical difference between the two paired samples and see the association between EGFR expression and EGFR promoter methylation changes. Meanwhile, we examined whether the differentially methylated DMRs were correlated with its clinical characteristics.

In the present study, we did sequenom analysis in two regions coincided with the CpG Island of EGFR promoter and identified that 7 CpG sites of Region I and 15 CpG sites of Region II were generally hypermethylated in malignant samples than in normal tissues. Both Region I and Region II were about 1000 nt far from the transcription initiation site in the 5′ UTR, and Region II was a part of the CGI3 with 192 CpG sites in the EGFR promoter. Among our 30 patients, more than 90% were hypermethylated in both two regions. The hypermethylation at EGFR promoter in gastric cancer was first detected in our study. Combined the data of relative EGFR expression, we found a significantly association between expression and methylation changes, which showed hypermethylation as an explanation of the stable maintenance of EGFR overexpression in gastric cancer. When overexpressed and activated, EGFR initiates a complex intracellular signal transduction cascade promoting cancer cell proliferation, apoptosis, angiogenesis and metastasisis.

Unlike that hypermethylation usually causes gene silencing in many situations, the biological basis and mechanisms that allow for the hypermethylation of promoter to cause overexpression of genes are still largely unknown. A possible explanation is that methylation of promoter region near the transcription start site leads to three-dimensional changes in the conformation of chromatin in this area, resulting in increased transcription^28^. Alternatively, methylation could prevent binding of repressors that normally prevent gene expression in normal cells.

Loeb, D. M. *et al.* showed that Wilms’ Tumor Suppressor Gene (WT1) is expressed in primary breast tumors despite tumor-specific promoter methylation^29^. Kelavkar, U. P. *et al.* found that hypermethylation of a specific CpG in prostate cancer cells resulted in transcriptional upregulation of 15-LO-1^30^. Ideraabdullah, F. Y. *et al.* explained the mechanism of genomic imprinting mediated by insulators as is present at the H19/Igf2 locus^31^. The model of imprinting regulation is that binding of insulator protein CCCTC-binding factor (CTCF) to the unmethylated ICR/DMD (designated imprinting center 1/ differentially methylated domain) prevents downstream enhancers from activating Igf2, leaving them available to activate transcription at H19; but for the methylated ICR/DMD, CTCF is unable to bind and resulting in expression of Igf2 while H19 is silenced^32^,^33^. In our study, we found one CTCF binding site (chr7: 55,085,278-55,085,406) was very close to our DMR1 (chr7: 55,085,467-55,085,911), whose methylation could abolish binding and silencing^34^. Thus, the Region I we detected may be an enhancer element/region. Future studies and identification of such repressors could lead to novel treatments that inhibit EGFR in cancer.

We also found that the methylation levels in Region I_⊿CpG_2 of the EGFR promoter were associated with the presence of distant metastasis, representing an unfavourable prognostic factor. Thus suggested that evaluation of high methylation levels of EGFR may be useful in identifying high-risk gastric cancer patients who eligible for multimodal treatments.

In summary, we found hypermethylation at the EGFR promoter in gastric cancer, which could be one of the mechanisms for high expression level of EGFR in gastric cancer. Consequently the methylation levels of EGFR could be considered as a potential epigenetic biomarker for gastric cancer status and progression.

## Author Contributions

L.H. and Y.L. supervised the experiment. X.W. and H.Z. designed the experimental protocol and carried out the experiment. Y.L., X.W., H.Z., J.Y., M.K., F.L., T.W. J.D. and Y.T. analyzed and discussed the experimental results. Finally, X.W. and Y.L. wrote the manuscript. All authors reviewed the manuscript.

## Additional Information

**How to cite this article**: Weng, X. *et al.* Hypermethylated Epidermal growth factor receptor (EGFR) promoter is associated with gastric cancer. *Sci. Rep.* **5**, 10154; doi: 10.1038/srep10154 (2015).

## Figures and Tables

**Table 1:** Clinicopathological parameters of the study participants.

**Clinicopathological parameters**	**Number**	**No. of cases (%)**
Age (years)		
≥ 60	21	70.00%
< 60	9	30.00%
Gender		
Male	23	76.67%
Female	7	23.33%
Histology differentiation		
Well	8	26.67%
Moderate	9	30.00%
Low	13	43.33%
Histology		
Tubular adenocarcinoma	17	56.67%
Mucinous adenocarcinoma	1	3.33%
Signet ring cell carcinoma	1	3.33%
Poorly differentiated adenocarcinoma	10	33.33%
Undifferentiated carcinoma	1	3.33%
Infiltration		
T1 + T2	11	36.67%
T3 + T4	19	63.33%
TNM stage		
I + II	4	13.33%
III + IV	26	86.67%
Distant metastasis		
Yes	3	10.00%
No	27	90.00%

**Table 2:** The difference between the methylation patterns (%) in paired tissues.

**CpGs**	**Normal**	**Tumour**	**P Value (Student paired t-tests)**	**P Value (Wilcoxon signed-rank test)**
Region I_CpG_2	68.13 ± 6.78	75.67 ± 16.22	2.25E−02	1.40E−03
Region I_CpG_3	78.80 ± 7.65	90.57 ± 6.33	6.51E−08	1.63E−05
Region I_CpG_4	55.92 ± 20.41	71.13 ± 11.53	5.00E−03	8.00E−03
Region I_CpG_5	40.00 ± 8.12	61.43 ± 12.19	1.19E−08	6.33E−06
Region I_CpG_6.7	59.83 ± 8.37	81.03 ± 7.72	1.78E−10	2.59E−06
Region I_CpG_8	53.60 ± 8.79	76.97 ± 9.28	1.26E−12	3.00E−06
Region I_DMR	59.38 ± 13.23	76.13 ± 9.74	2.77E−10	1.30E−08
Region II_CpG_1	31.27 ± 8.22	54.13 ± 8.94	5.85E−10	3.33E−06
Region II_CpG_2	37.20 ± 8.69	49.83 ± 10.88	3.71E−06	2.72E−05
Region II_CpG_3	28.07 ± 6.42	35.23 ± 9.54	1.00E−03	8.00E−04
Region II_CpG_4	17.17 ± 3.77	22.97 ± 9.45	1.80E−03	1.80E−03
Region II_CpG_5.6.7	23.30 ± 5.03	33.90 ± 10.53	2.41E−05	1.19E−05
Region II_CpG_8.9	12.56 ± 5.64	23.80 ± 12.02	4.00E−04	3.00E−04
Region II_CpG_11	27.80 ± 4.32	33.97 ± 6.27	6.82E−05	5.98E−05
Region II_CpG_12	19.77 ± 5.33	24.20 ± 7.69	9.30E−03	1.84E−02
Region II_CpG_13.14	21.50 ± 4.12	24.77 ± 7.64	3.67E−02	6.23E−02
Region II_CpG_15	19.77 ± 5.33	24.20 ± 7.69	9.30E−03	1.84E−02
Region II_CpG_16	19.13 ± 5.93	19.53 ± 7.94	8.13E−01	8.71E−01
Region II_CpG_17	27.80 ± 4.32	33.97 ± 6.27	6.82E−05	5.98E−05
Region II_CpG_18	9.73 ± 2.60	9.03 ± 2.62	2.24E−01	2.73E−01
Region II_DMR	22.70 ± 7.63	29.96 ± 12.18	2.17E−05	3.15E−07

**Table 3:** Correlation of participant characteristics and the methylation differences in DMR I and DMR II.

	**DMR I**		**DMR II**	
**Parameters**	**r**^**2**^	**P value**	**r**^**2**^	**P value**
Age	0.0165	0.4991	0.0117	0.5689
Gender	0.0075	0.6495	0.0458	0.2562
Histology differentiation	0.0007	0.8878	0.0038	0.7462
Histology	0.0021	0.8111	0.0004	0.9185
Infiltration	0.0268	0.3870	0.0323	0.3416
TNM stage	0.0104	0.5919	0.0128	0.5511
Distant metastasis	0.0346	0.3247	0.0396	0.2918

**Table 4:** The association between the methylation and clinic pathological factors.

**Sites**	**Age**	**Gender**	**Histology differentition**	**Histology**	**Infiltration**	**TNM stage**	**Distant metastasis**
Region I_⊿CpG_2	0.9990	0.1970	0.1964	0.3794	0.5463	0.7043	0.0323 *
Region I_⊿CpG_3	0.5010	0.3100	0.4170	0.4530	0.6440	0.3530	0.8810
Region I_⊿CpG_4	0.3360	0.6310	0.8240	0.6820	0.8370	0.4960	0.3130
Region I_⊿CpG_5	0.4130	0.6410	0.5280	0.4220	0.4960	0.8530	0.6430
Region I_⊿CpG_6.7	0.6630	0.7590	0.5160	0.6270	0.8710	0.5160	0.5420
Region I_⊿CpG_8	0.7310	0.8190	0.9890	0.7950	0.4720	0.8350	0.8170
Region II_⊿CpG_1	0.3661	0.2595	0.4018	0.5173	0.8118	0.5143	0.6482
Region II_⊿CpG_2	0.5710	0.4390	0.5940	0.6000	0.8340	0.2690	0.9470
Region II_⊿CpG_3	0.6280	0.0652	0.2126	0.2959	0.9243	0.1801	0.9632
Region II_⊿CpG_4	0.6200	0.6200	0.2160	0.2910	0.5840	0.3310	0.8400
Region II_⊿CpG_5.6.7	0.6040	0.3520	0.7240	0.7330	0.8570	0.3200	0.5800
Region II_⊿CpG_8.9	0.5210	0.1550	0.7450	0.3560	0.2670	0.6660	0.2220
Region II_⊿CpG_11	0.2520	0.7030	0.2680	0.6190	0.5590	0.2900	0.6390
Region II_⊿CpG_12	0.7880	0.5780	0.3080	0.3770	0.6600	0.4730	0.2120
Region II_⊿CpG_13.14	0.8300	0.8070	0.1910	0.5000	0.9580	0.3430	0.6090
Region II_⊿CpG_15	0.7880	0.5780	0.3080	0.3770	0.6600	0.4730	0.2120
Region II_⊿CpG_16	0.9050	0.8460	0.1110	0.3090	0.4070	0.3670	0.3150
Region II_⊿CpG_17	0.2520	0.7030	0.2680	0.6190	0.5590	0.2900	0.6390
Region II_⊿CpG_18	0.3520	0.1500	0.3770	0.7290	0.1240	0.3300	0.4990

^*^P < 0.05
